# Does the Clock Tick Slower or Faster in Parkinson’s Disease? – Insights Gained From the Synchronized Tapping Task

**DOI:** 10.3389/fpsyg.2018.01178

**Published:** 2018-07-11

**Authors:** Shin-ichi Tokushige, Yasuo Terao, Shunichi Matsuda, Toshiaki Furubayashi, Takuya Sasaki, Satomi Inomata-Terada, Akihiro Yugeta, Masashi Hamada, Shoji Tsuji, Yoshikazu Ugawa

**Affiliations:** ^1^Department of Neurology, Graduate School of Medicine, The University of Tokyo, Tokyo, Japan; ^2^Department of Cell Physiology, Kyorin University, Mitaka, Japan; ^3^Department of Rehabilitation, Faculty of Medical Science and Welfare, Tohoku Bunka Gakuen University, Sendai, Japan; ^4^Department of Neurology, School of Medicine, Fukushima Medical University, Fukushima, Japan

**Keywords:** Parkinson’s disease, temporal integration, synchronized tapping, basal ganglia, internal clock

## Abstract

The rhythm of the internal clock is considered to be determined by the basal ganglia, with some studies suggesting slower internal clock in Parkinson’s disease (PD). However, patients may also show motor hastening when they walk (festination) or are engaged in repetitive tapping, indicating faster ticking of the internal clock. Is the internal clock slower or faster in PD? The purpose of this study was to answer this question, i.e., how fast and slow a rhythm they can synchronize with, especially with reference to the limit of sensorimotor synchronization or temporal integration, representing the threshold of slower pace they can entrain into their motor actions, which is known to lie between 2 and 3 s in normal subjects but not yet studied in PD. We employed a synchronized tapping task that required subjects to tap the key in synchrony with repetitive tones at fixed interstimulus intervals (ISI) between 200 and 4800 ms. Twenty normal subjects and sixteen PD patients were enrolled, who were classified into early and advanced PD groups by UPDRS-III (early: 15 or less, advanced: more than 15). The ISI at which the response changes from synchronizing with the tones to lagging behind them was considered to be the limit of temporal integration. Early PD patients responded ahead of the tones (negative asynchrony), which became more apparent with repeated tapping. This suggested “faster” ticking clock even in the presence of the pacing tones. In normal subjects, the limit of temporal integration was around 2–3 s: below this, subjects could synchronize with the tones, while above it they had difficulty in synchronization. In early PD patients, the limit of temporal integration was significantly longer than in normal subjects, pointing to their enhanced ability to synchronize also with slower paces of tones, but advanced PD patients had significantly shortened limits, suggesting that advanced patients lost this ability. In conclusion, the limit of temporal integration is initially longer but gets shorter as the disease progresses. It can be explained by the hastening of the internal clock at the earlier stages of PD, followed by the loss of temporal integration.

## Introduction

Animal and human neuroimaging studies over the last 10–20 years have made it clear that the neural structures responsible for temporal processing are those closely associated with the motor system such as the basal ganglia and the cerebellum. One of the widely accepted theories of temporal processing, the scalar expectancy theory (SET) assumes an “internal clock” in the brain, an imaginary metronome-like pacemaker, along with a working memory, a reference memory, and a decision process (Gibbon et al., 1984; Church et al., 1994); to perceive the duration of time, the brain would “count” how many pulses the pacemaker ticks during the time interval to be measured and compares the count with the reference time duration stored in memory. Although there is as yet no evidence for the neural substrate of a dedicated pacemaker within the central nervous system, SET has been successful in explaining many behavioral aspects of temporal processing as studied by tasks addressing time perception and production (Buhusi and Meck, 2005). By this account, if the internal clock ticks faster, the interval of time is perceived as longer, because more beats of pacemaker would tick during the same interval, whereas when the subjects are required to produce a verbally dictated time interval (e.g., 3 s), the duration produced will be shorter, since the ticks are faster. Exactly the reverse would happen when the pacemaker slows down.

As the basal ganglia are considered to be responsible for temporal processing (Rao et al., 1997; Schubotz et al., 2000; Nenadic et al., 2003; Jones and Jahanshahi, 2009), patients with Parkinson’s disease (PD), a basal ganglia disorder, are expected to show various abnormalities of temporal processing. Among these, that of the internal clock rhythm has been especially studied in PD, since the rhythm of the internal clock is considered to be determined by the basal ganglia (Buhusi and Meck, 2005). In fact, PD patients underestimate the duration of given stimuli, and over-reproduce their durations (Pastor et al., 1992; Lange et al., 1995; Koch et al., 2008). The results are consistent with the notion that the rhythm of the internal clock is abnormally slowed in PD (Perbal et al., 2005), and intuitively understandable considering the overall slowness of their movement. The notion of slower internal clock is also consistent with the findings of studies in which animals treated with dopamine blockers performed the temporal generation task (Pastor et al., 1992; Lange et al., 1995; Koch et al., 2008).

Paradoxically, however, despite the overall slowness in their movement, PD patients are also known to exhibit motor hastening, as typically observed when PD patients walk; although they start out walking slowly, the speed sometimes tend to grow faster and faster (festination). A similar hastening is observed when patients are engaged in repetitive tapping, especially in PD patients with freezing of gait (Tolleson et al., 2015; Honma et al., 2016). The authors suggested that this motor hastening may be the result of the internal clock to operate faster, rather than slower, when engaged in a repetitive action.

In order to study whether PD patients are slower or faster in their rhythm generation, we considered it important to investigate spontaneous motor tempo of PD patients as a measure of their internal clock speed, particularly in association with motor action, whereas many previous studies have used tasks addressing time perception and tempo processing (see Jones, 1976; Fraisse, 1984; Harrington and Haaland, 1999; Drake et al., 2000). Indeed, neurological disorders involving the basal ganglia or cerebellum has been shown to exhibit abnormal patterns of tapping performance (Yahalom et al., 2004; Matsuda et al., 2015).

In order to study the motor tasks involving tapping at a regular interval both in isolation or in synchrony with a regularly paced tone, for which both the basal ganglia and cerebellum play important roles (Ivry, 1996; Rao et al., 1997). O’Boyle et al. (1996) tested PD patients, on and off levodopa, repeatedly on a tapping task with a constant target duration (550 ms), whose pace had been established previously during an initial period of tapping in synchrony with the beats of a regular metronome (synchronization task). Then the same subjects were required to produce a regular series of self-timed inter-tap intervals (continuation task). The mean self-paced inter-response interval (IRI) of parkinsonian patients was generally shorter than that of controls (“faster tapping”), consistent with motor hastening with repeated motor action. According to the Wing and Kristofferson (1973) model, the authors partitioned the total variance (TV) into ‘clock’ variance (CV) and ‘motor-delay’ variance (MDV) attributable to hypothetical central ‘clock’ and more peripheral ‘motor-implementation’ processes. Values of TV, CV, and MDV in PD were all significantly higher when subjects were ‘off’ medication, indicating variance of both central and peripheral origin. During the ‘on’ medication condition, only CV was significantly higher than the control value, suggesting central origin of variance. Therefore, variability of tapping in PD could be decomposed both into central clock and peripheral motor implementation variability, although the results depended on the group of patients studied and differentially l-dopa on/off state of the patients. In contrast, for patients with cerebellar disorders, medial cerebellar lesions mainly showed peripheral motor variability clock, whereas lateral cerebellar lesions were associated central clock variability (Ivry, 1996).

Most importantly, although one or a couple of tapping tempos were investigated in these studies, tapping performance may largely differ according to the faster and slower paces of the tapping tempo (Claassen et al., 2013; Tolleson et al., 2015). Normal subjects can tap in synchrony or slightly in advance of tones up to a certain interval, i.e., at faster paces. When the intervals exceed 2–3 s, i.e., at slower paces, subjects can no longer predict the time of oncoming tone and synchronize their responses with the tones, and their responses eventually come to lag behind the tones, which are actually fulfilled by reactions to the tones (Mates et al., 1994; Claassen et al., 2013; Tolleson et al., 2015). Since subjects have to predict the time of each oncoming tone in their minds to perform this task, and must prepare each tap to occur just in time with the pace of the tones, this corresponds subjectively to the limit to the span of time one can integrate and perceive as a “perceptual unit,” i.e., that which one can grasp as a unit of time and prepare for. This is supported by a number of studies on the temporal reproduction of stimuli (Pöppel, 1997). For example, whereas stimuli up to 3 s long are reproduced relatively accurately in temporal reproduction tasks, longer stimuli are incorrectly reproduced (Pöppel, 1978, 1997). Similarly, the appearance of the Necker cube alternates, with its corner appearing to protrude anteriorly or posteriorly, at intervals of approximately 3 s (von Steinbüchel et al., 1996; Pöppel, 1997). These studies suggest that there is a limit to the time window within which the brain can integrate stimuli into a perceptual unit, representing a “subjective present” (Pöppel, 1997), and that the limit of temporal integration can be pragmatically defined using a synchronized tapping task (Mates et al., 1994; Pöppel, 1997; Repp, 2005). The interval at which the transition from synchronized to delayed tapping occurs represents the limit of temporal integration. After this ISI limit, the subjects’ tapping comes to lag behind the tone; although the subjects are still trying to synchronize the tapping with the tones, the taps are actually made in response to the tones. Considering that the tapping performance varies drastically from a certain ISI, it may be difficult to explain the results of tapping task for all ISIs using a single model (Wing and Kristoffersen model). Furthermore, in a study which measured the cerebral blood flow (CBF) of normal subjects performing rhythmic repetitive finger movements by positron emission tomography, the pattern of CBF change depended on the frequencies of movements (Sadato et al., 1996). This is another evidence that distinct cortical and subcortical regions are engaged differentially in performing synchronized tapping tasks at short and long ISIs, and that the performances of taping task for all ISIs may not necessarily be explained by a single model.

The limit of temporal integration is usually around 3 s (Pöppel, 1997), and we predicted that temporal integration, considered to be one type of the time processing in the suprasecond order, would be affected in basal ganglia disorders such as PD; it has been suggested that the basal ganglia are involved in temporal processing in the suprasecond range, whereas for processing shorter time intervals in the millisecond range, the cerebellum plays a greater role, and this should allow us to investigate the range of rhythm generation (Hazeltine et al., 1997). In this context, to investigate the abnormality of the internal rhythm generation in PD, it was considered important to use the synchronized tapping task in which the subject taps a key in synchrony with a sequence of repetitive tones presented at a fixed interstimulus interval (ISI) (sensorimotor integration or temporal integration), where the ISI were from 300 to 4800 ms in different sessions (Mates et al., 1994; Takano and Miyake, 2007). Thus, we investigated not only how fast a pace the patients can synchronize with, but also the lower limit of the pace of tones (longer ISIs) with which the subjects can synchronize their tapping with, corresponding to the limit of temporal integration; if a subject can tap in synchrony with the tones, this means that the subject can generate a rhythm in pace with the rhythm of the tones.

The present study aimed to investigate the limit of temporal integration in PD patients and to relate this limit to the pace of the internal clock. More specifically, we predicted that, if the internal clock of PD patients is “ticking” more slowly, they would be able to synchronize with slower paces only and not faster paces of the tone. Since their slower internal clock would allow only slower tapping, patients would tend to lag behind the tones with repeated tapping at faster paces (shorter ISIs). To our knowledge, there has been no study which investigated the abnormal limit of temporal integration in PD patients.

In view of the notion that the basal ganglia are involved in temporal processing in the suprasecond range (Rao et al., 1997; Schubotz et al., 2000; Nenadic et al., 2003; Jones and Jahanshahi, 2009), we hypothesized that temporal processing in the second range would be disrupted in PD patients, i.e., the main abnormality of temporal processing would lie within the suprasecond range. We predicted that PD patients would perform worse than normal subjects in that they show more delayed tapping compared to normal subjects, reflecting slower internal clocks or increased variability in tapping performance. Thus the limit of temporal integration would be shorter than that of normal subjects. Although this is a behavioral study and no neuroimaging or pathological evidence is available, it may contribute to the understanding of the physiological aspect of temporal processing in PD.

## Materials and Methods

### Temporal Integration of PD Patients and Normal Subjects

#### Subjects

Sixteen PD patients and twenty age-matched normal subjects were enrolled. The patients were classified into early (UPDRS-III ≤ 15) and advanced PD groups (UPDRS-III > 15). We divided the patients depending on their UPDRS-III score. We set the cut-off point at 15, and divided the patients into those with UPDRS-III score above and below this cut-off point, since 15 was the closest round number to the median of UPDRS-III scores of all patients. The characteristics of subjects are shown in **Table 1**. All patients were studied in the clinically ON state, approximately 2 h after taking anti-parkinsonian drugs.

**Table 1:** Characteristics of normal subjects and Parkinson’s disease (PD) patients enrolled in the tapping and reaction time tasks.

	Normal	Early PD	Advanced PD	*p-*value
Number	20	9	7	–
Male: Female	9:11	6:3	3:4	–
Age	71.5 ± 9.0	68.7 ± 9.0	75.6 ± 6.4	0.29
(years, mean ± *SD*)				
MMSE	27.6 ± 2.5	27.3 ± 2.6	27.1 ± 2.7	0.93
(mean ± *SD*)				
Disease duration	–	6.9 ± 3.8	7.0 ± 5.9	0.96
(years, mean ± *SD*)				
UPDRS-III	–	6.4 ± 3.5	25.4 ± 10.2	<0.001
(mean ± *SD*)				
Levodopa equivalent	–	452 ± 232	310 ± 194	0.22
dose (mg/day, mean ± *SD*)				


We obtained written informed consent from all subjects prior to the experiments. The experimental procedures were approved by the Ethics Committee of the University of Tokyo, and the study was conducted in accordance with the ethical standards of the Declaration of Helsinki.

#### Synchronized Tapping Task

**FIGURE 1:**
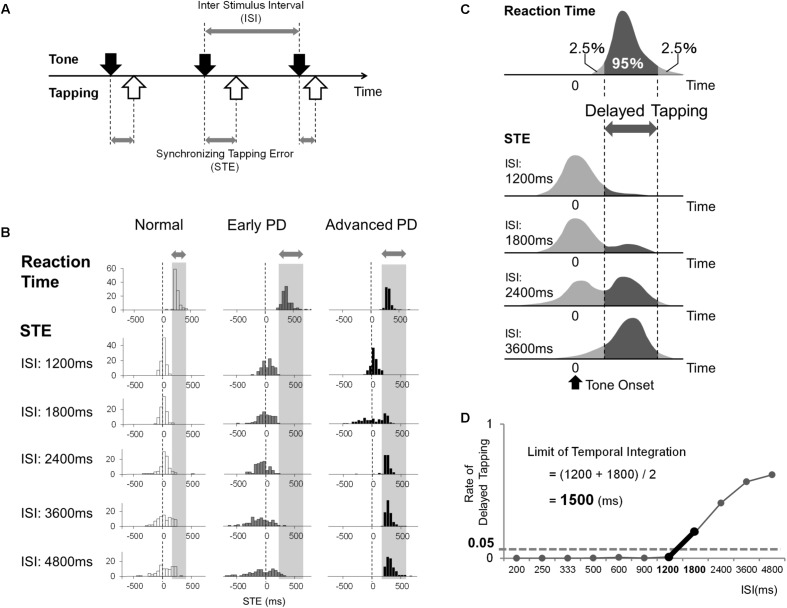
**(A)** The subjects tapped the button in synchrony with the sequences of auditory tones presented at fixed interstimulus intervals (ISIs) between 200 and 4800 ms. We measured the time difference [synchronizing tapping error (STE)] between the times of taps (white arrows) and those of tones (black arrows). **(B)** Examples of the distributions of reaction time and STE in a normal subject, early PD patient, and advanced PD patient. The gray bar indicates the 95% area of the subject’s reaction time. **(C)** The schematic distributions of a subject’s reaction time and STE at each ISI. When the ISI is short enough, the distribution of STE has a single peak. However, as the ISI gets longer, the distribution of STEs becomes broader, and another peak appears that corresponds to the range of reaction time, which means that the subject taps after hearing the tone (delayed tapping) instead of synchronizing. We estimated the rate of delayed tapping at each ISI by calculating the rate of STE that corresponded to the 95% area of the subject’s reaction time. **(D)** The method used to calculate the limit of temporal integration of each subject.

The synchronized tapping task is depicted in **Figure 1A**. One of the buttons of a USB game controller (Microsoft Sidewinder Plug and Play Gamepad, Microsoft, Seattle, WA, United States) was used as the response key. The experiment was performed using a personal computer (Dell Inc., Texas, United States) and a software (Experiment Builder, SR Research, Ontario, Canada), which enables presentation of sounds and collection of the time of button press made by the subjects, as described in the previous paper of Matsuda et al. (2015). The subject’s task was to tap the button precisely in synchrony with the tones presented at a fixed interstimulus interval (ISI) through a speaker. In each session, the ISI is fixed at one value, and typically 110 trials (taps) are performed per session. The ISI in each sequence was kept at a constant value (isochronous sequence), selected from 11 different ISIs according to our previous paper (Matsuda et al., 2015): 200, 250, 333, 500, 600, 900, 1200, 1800, 2400, 3600, and 4800 ms. The order of presentation of the sequences with different ISIs was randomized across subjects, and each subject eventually completed the sessions for all ISIs. In each session, sequences consisting of 110 tones were presented. Each tone was a simple tone with a frequency of 500 Hz and duration of 50 ms.

Synchronizing tapping error (STE) refers to the time of the tap relative to the time of the tone. The STE, as a measure of the abnormal speed of the internal clock, should reveal two aspects of abnormal temporal processing: impaired rhythm generation and variability (inaccuracy) in rhythm generation. The former is expected to manifest itself in the deviation of tapping time relative to the time of tone as tapping proceeds repetitively (deviation of STE), whereas the latter is expected to manifest itself in the variability of tapping with respect to the rhythm of the tone (variability in STE). In normal subjects, although the sound level was not measured, it was at a fixed level mimicking that of a metronome, and not as loud to be disturbing to the subjects. The experiments were performed in a quiet room with low ambient noise, whose door was closed to shut out other people coming into the room during the experiments. There was no feedback sound to the button press; in other words, the computer was not programmed to make any specific sound in response to the key press. However, the subjects were able to hear the slight click noise emitted from the key. The subjects were instructed to use the index finger to tap the button.

#### Simple Reaction Task

A good synchronization performance accompanies negative asynchrony delay, in which tapping precedes the time of the tomes approximately by 30–50 ms, because to achieve exact synchrony of tapping with the tones the subjects should start ahead of the timing of the tone, where a slight overestimation of the movement time is known to occur even in normal subjects (Mates et al., 1994; Aschersleben and Prinz, 1995). However, when synchronization fails, the subjects’ tap tend to lag behind the tones (see section “Introduction”).

To parse the tapping performance into synchronized and delayed tapping, in addition to the synchronized tapping task, we also implemented the simple reaction time task in a separate session, in which subjects tapped the button in response to the tones, presented at a random interval, rather than synchronizing with them. We employed this experiment to obtain the range of simple reaction time to simple tones in each subject, which is necessary to judge if a subject’s response to a tone was ‘synchronized to the sound’ or was actually made ‘in response to’ the tones after hearing the sound’ (in other words, delayed tapping). In the tapping task, if the time of tapping delay from the sound fell into to the range of the subject’s reaction time range (in this paper, 95 percentile of the subject’s reaction time), the tap can be regarded as actually made in response to the tones, indicating that, although the subjects tried to tap in synchrony with them, synchronized tapping was impossible for the subject. In such a situation, the interstimulus interval has exceeded the subject’s limit of temporal integration, and the subjects resort to reactive “delayed” tapping, though unconsciously.

In this experiment, the interstimulus interval was randomly selected from 2400 to 4800 ms, with a increment intervals of 200 ms (2400 ms, 2600 ms, 2800 ms,…. 4800 ms). This interval was randomly selected every time the subject tapped the button, such that the time of upcoming tone could not be predicted by the subject. The total number of sounds was 110 times.

#### Data Collection and Statistical Analysis

The data collected were button press times and tone presentation. To see the time course of tapping performance, all the 110 taps were analyzed. In calculating the limit of temporal integration, the taps before the ninth sound were ignored. In the synchronized tapping task, the next ten additional taps were also ignored, to see the plateau of tapping performance. From these data, we derived the time difference between each tap onset and stimulus onset [synchronizing tapping error (STE)], according to the methods of Mates et al. (1994). STE was measured from the stimulus onset to the time of tap onset, where a positive value indicated that the tap was delayed relative to the tone, while a negative value indicated that the tap preceded the tone (**Figure 1A**).

The PD patients nearly always produced only one tap per each sound, following our instruction. For all subjects, each tap could reliably associated with the corresponding tone, by finding out the closest tone in relative time, from the time preceding the tone by half the ISI to the time following the tone by half the ISI. Thus we had no trouble in calculating STE.

The data is first depicted in the form of histograms relative to the time of the tone at each ISI (**Figure 1B**). The abscissa shows the STE, that is, the time of the tap relative to the time of the tone. This is plotted against the proportion of trials at each bin on the ordinate. The vertical dashed line (0 ms line) in each figure shows the time of the tone. When STE is negative, the taps occurred before the corresponding tone; when positive, the taps lagged behind the tones. Based on the reaction time and STE data, the limit of temporal integration was calculated for each subject. **Figure 1C** shows schematic illustrations of the distributions of the reaction time (top histogram) and STE plotted as a histogram as described above. At shorter ISIs, normal subjects were able to tap approximately in synchrony with the tones, as shown by the distribution clustered around 0 ms (first histogram for STE in **Figure 1C**). Above a certain ISI, subjects were no longer able to tap in synchrony with the tones, and the proportion of taps that lagged behind the tones increased (second and third histograms). As the ISI gets longer, therefore, the subject had more difficulty synchronizing the taps with the tones, and the STE began to show a broader and delayed distribution, indicating that the subject is beginning to tap after the tones rather than with accurate synchronization. Actually, when the taps lag behind, they occur at a latency of 100–200 ms after the tone, finally forming a relatively sharp peak in the histogram (fourth histogram for STE in **Figure 1C**, see also Matsuda et al., 2015). Therefore, there is a transition from a state in which subjects predict the time of the next tone and tap in anticipation to a state in which subjects are actually reacting to the tones.

We defined “delayed tapping” as taps with latency exceeding the 95% percentile of each subject’s reaction time distribution (**Figure 1C**). We plotted the proportion of delayed tapping for each ISI, and the limit of temporal integration was defined as the ISI where the proportion of delayed tapping exceeded 5% of the total trials. For this particular subject (**Figure 1D**), the proportion of trials with delayed tapping was less than 5% when the ISI was under 1200 ms, while it exceeded 5% when the ISI was greater. This suggests that the proportion of delayed tapping reached the threshold of 5% between ISIs of 1200 and 1800 ms. Accordingly, we estimated the limit of temporal integration to be 1500 ms, i.e., the midpoint between 1200 and 1800 ms.

#### Statistical Assessment

The following statistical analyses were performed using a commercial software (version 19.0; SPSS Inc., Chicago, IL, United States). (i) To compare the reaction times between early and advanced PD patients and normal subjects, analysis of variance (ANOVA) was used. (ii) To assess how accurately the subjects reproduced ISIs of the presented tone sequence by tapping, the average STE at each ISI was calculated for each group. This parameter was analyzed using ANOVA with a between-subject factor of subject group. (iii) To compare the limit of temporal integration among groups, ANOVA was performed with a between-subject factor of subject group. If this result was significant, Tukey’s *post hoc* test was performed to identify which of the pairs showed significant differences. For all analyses, the significance threshold was set at *p* < 0.05.

To investigate whether age, disease severity, disease duration, or levodopa equivalent dose (LED) influenced the limit of temporal integration, multiple linear regression analyses were performed for PD patients. We took the limit of temporal integration as the outcome variable, and age, disease duration, and LED as predictor variables. The partial regression coefficients of predictor variables were expressed as β, and the *p*-value from the *t*-test for the regression slope of predictor variables was used to determine the probability.

Since the experiment was performed with subjects in the L-Dopa ON state, it was necessary to confirm that the LED did not affect the comparison of temporal integration among patient groups. Thus we performed analysis of covariance (ANCOVA), taking the limit of temporal integration as the dependent variable, the category of disease severity (early or advanced) as the independent variable, with LED as the covariate.

### The Effect of L-Dopa on Synchronization

#### Subjects

Nine PD patients were enrolled (**Table 2**). Eight patients were taking anti-parkinsonian drugs, and were asked to visit our laboratory when they were in the clinically OFF state (at least 5 h after the last L-Dopa dose), while one patient had never taken any anti-parkinsonian drugs before.

**Table 2:** Characteristics of PD patients enrolled in the L-dopa ON/OFF tapping task.

Number	9
Male: Female	5:4
Age (years, mean ± *SD*)	71.9 ± 9.4
MMSE (mean ± *SD*)	27.8 ± 1.6
Disease duration (years, mean ± *SD*)	1.4 ± 1.3
UPDRS-III (mean ± *SD*)	25.7 ± 15.0


#### Synchronized Tapping Task Before and After Taking L-Dopa

Subjects performed the synchronized tapping task once as a baseline trial. Then they took 200 mg L-Dopa with benserazide (Madopar^®^, Chugai Pharma, Tokyo, Japan), and performed the task again 90 min later. Their UPDRS-III scores were also assessed before and after intake. The same computer, game controller and software as the synchronized tapping task were used in this experiment.

#### Data Collection, Statistical Analysis, and Statistical Assessment

To examine the time course of tapping performance, all the 110 taps were analyzed. On the other hand, for evaluating the difference between L-Dopa On and Off, the taps before the ninth sound and additional 10 taps were ignored, to evaluate the plateau of tapping performance. We calculated the subjects’ STE at each ISI both before and after taking L-Dopa. We examined whether the STE at each ISI was significantly altered by L-Dopa by means of the paired *t*-test. For all analyses, the significance threshold was set at *p* < 0.05.

## Results

Sample distributions of the reaction times and STEs for a normal subject, an early PD patient, and an advanced PD patient are depicted in **Figure 1B**.

**Figure 1B** shows representative histograms depicting the proportion of trials plotted against STE, i.e., the time of tapping relative to the time of tone (shown as the vertical dashed line at 0 ms) at various ISIs in a normal subject (left column) and early and advanced PD patients (middle and right columns).

The normal subject (left column in **Figure 1B**) was able to tap approximately in synchrony with the tones presented at ISIs of up to 2400–3600 ms, although the tapping became more variable around the time of the tones as ISI increased. This is reflected in the lowering of the height along with the broadening of the peak in the histograms as the ISI increases, presumably reflecting the scalar property of time processing, with the peak of distribution staying approximately around 0 ms. At ISI 4800 ms, however, the distribution begins to lag behind. In the reaction time task, the response time to a single auditory stimulus was measured (top row). The distribution of STE was similar to the reaction time distribution (top histogram).

**FIGURE 2:**
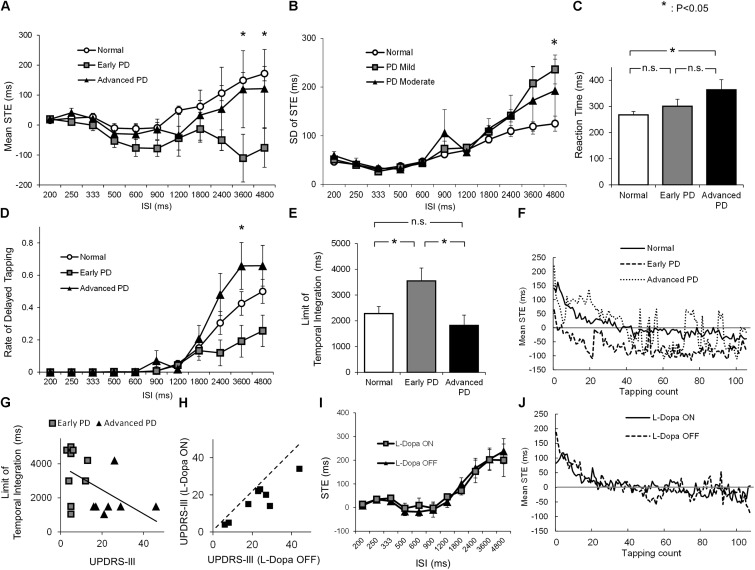
**(A)** The average STE of normal subjects (white dot), early PD patients (gray rectangle), and advanced PD patients (black triangle) for each ISI (mean ± standard error). **(B)** The standard deviation (SD) of STE of normal subjects (white dot), early PD patients (gray rectangle), and advanced PD patients (black triangle) for each ISI (mean ± standard error). **(C)** The average reaction time of normal subjects (white) and early (gray) and advanced PD patients (black). **(D)** The average rate of delayed tapping of normal subjects (white dot), early PD patients (gray rectangle), and advanced PD patients (black triangle) for each ISI (mean ± standard error). **(E)** The average length of the limit of temporal integration in normal subjects (white), early (gray) and advanced PD patients (black), calculated from the STE and reaction time. **(F)** The time course of mean STE of normal subjects, early PD patients, and advanced PD patients, when the ISI was 900 ms. **(G)** The scatter plot of UPDRS-III score and the limit of temporal integration in early (gray rectangle) and advanced (black triangle) PD patients. **(H)** The UPDRS-III scores of PD patients before (OFF) and after (ON) taking L-Dopa 200 mg. **(I)** The average STE of patients in the L-Dopa OFF state (black triangle) and ON state (gray rectangle) for each ISI (mean ± standard error). **(J)** The time course of mean STE of the PD patients when L-Dopa was ON and OFF when the ISI was 900 ms. Asterisks indicate significant difference (*p* < 0.05), and n.s. indicates “not significant” (*p* ≥ 0.05).

**Figure 1B** (middle column) shows that this early PD patient (with UPDRS-III ≤ 15) tapped well ahead of the tones (negative asynchrony); the distribution of STE took a negative value across trials at ISI 1200–4800 ms, which implied that they tended to tap ahead of the tones in more trials than normal subjects. Although the width of each histogram broadened with increasing ISI, it did not broaden significantly more than those of normal subjects, since the standard deviation of STE (**Figure 2B**) showed a significant difference among the three groups only when ISI was 4800 ms (one-way ANOVA, *p* = 0.025), and not when ISI was shorter than 4800 ms. At ISIs longer than 4800 ms, however, the proportion of trials in which this subject’s tapping lagged behind the tones increased, as did those of normal subjects. Nevertheless, the number of taps lagging behind the tones was actually lower in this patient than in normal subjects, as depicted by the smaller peak lagging behind the time of tones at the longer ISIs. Across early PD patients, this trend toward negative asynchrony emerged in some patients at ISIs as high as 1200 ms, while for some patients, the STE stayed at a negative value even at ISIs of 2400–4800 ms (not shown). For more advanced PD patients, in contrast, with UPDRS-III of > 15 (**Figure 1B**, right column), although negative asynchrony was noted at ISI 1200 ms, the tapping consistently lagged behind the tone (positive STE values around 200 ms) at ISIs greater than 1800–2400 ms.

To see how the tapping performance of normal subjects and early and advanced PD patients changed with increasing ISI, we plotted the STE (ordinate) as a function of ISI (abscissas, **Figure 2A**). In normal subjects (**Figure 2A**, white dots), the STE was very close to 0 or slightly negative up to ISIs of 900 ms, but tended to increase thereafter. This increase corresponds to the taps of normal subjects gradually beginning to lag behind the tones at larger ISIs. In early PD patients (gray squares), in contrast, the STE gradually decreased below zero, deviating toward a more negative value with some variability, as ISI increased (negative asynchrony). When the ISI was longer than 2400 ms, STEs decreased in early PD patients, whereas in normal subjects and advanced PD patients, STEs increased after ISI 900 ms. As a result, the STEs of early PD patients began to deviate from those of the two other groups (normal subjects and advanced PD patients) at longer ISIs.

To confirm this observation, we calculated the average STE for each ISI. For ISIs shorter than 2400 ms, there was no significant difference in the STEs among the three groups [ISI 200 ms, *F*_0.05_ (2,33) = 3.28, *F* = 0.062, *p* = 0.94; ISI 250 ms, *F* = 1.67, *p* = 0.20; ISI 333 ms, *F* = 1.26, *p* = 0.30; ISI 500 ms, *F* = 1.24, *p* = 0.30; ISI 600 ms, *F* = 2.25, *p* = 0.12; ISI 900 ms, *F* = 2.30, *p* = 0.12; ISI 1200 ms, *F* = 0.31, *p* = 0.73; ISI 1800 ms, *F* = 0.204; *p* = 0.38; ISI 2400, *F* = 2.21, *p* = 0.13]. A significant difference was detected among the three groups at ISIs 3600 and 4800 ms (ISI 3600 ms, *F* = 5.03, *p* = 0.012; ISI 4800 ms, *F* = 5.10, *p* = 0.012). Tukey’s *post hoc* test showed that the STE of early PD patients was significantly smaller than that of normal subjects at ISIs 3600 ms (*p* = 0.010) and 4800 ms (*p* < 0.010). Yet there was no significant difference in STE between normal subjects and advanced PD subjects at these ISIs (ISI 3600 ms, *p* = 0.94; ISI 4800 ms, *p* = 0.83). Although the difference between early and advanced PD patients did not reach significance, it approached a trend (ISI 3600 ms, *p* = 0.086; ISI 4800 ms, *p* = 0.12).

As mentioned above, the standard deviation of STE showed a significant difference only when ISI was 4800 ms (**Figure 2B**, ISI 200 ms, *F* = 1.05, *p* = 0.36; ISI 250 ms, *F* = 0.063, *p* = 0.94; ISI 333 ms, *F* = 0.38, *p* = 0.68; ISI 500 ms, *F* = 0.39, *p* = 0.68; ISI 600 ms, *F* = 0.090, *p* = 0.91; ISI 900 ms, *F* = 1.26, *p* = 0.30; ISI 1200 ms, *F* = 0.26, *p* = 0.77; ISI 1800 ms, *F* = 1.12; *p* = 0.34; ISI 2400, *F* = 1.17, *p* = 0.32; ISI 3600, *F* = 2.69, *p* = 0.083; ISI 4800 ms, *F* = 4.16, *p* = 0.025). This means that the accuracy of tapping tempo is not very different between normal subjects and PD patients.

**Figure 2C** compares the reaction times of the three groups, which were significantly different from each other (*F* = 4.43, *p* = 0.026). As expected, advanced PD patients showed a significantly longer reaction time than normal subjects (Tukey’s *post hoc* test, *p* = 0.015), whereas there was no significant difference between normal subjects and early PD patients (*p* = 0.51).

To assess the limit of temporal integration among the three groups, **Figure 2D** shows the proportion of delayed tapping at each ISI, estimated using the STE and reaction time. For ISIs longer than 1800 ms, early PD patients tended to show a smaller amount of delayed tapping than the other two groups, although the difference was statistically significant only at ISI 3600 ms (*F* = 4.06, *p* = 0.026). Tukey’s *post hoc* test showed that the proportion of delayed tapping in early PD patients was significantly smaller than that in advanced PD patients (*p* = 0.021), whereas no significant difference was found between normal subjects and early PD patients (*p* = 0.19) or between normal subjects and advanced PD patients (*p* = 0.25).

Consistent with this finding, the limit of temporal integration (**Figure 2E**) was significantly different among the three groups (*F* = 4.39, *p* = 0.020); the limit was significantly longer in early PD patients than in normal subjects (Tukey’s *post hoc*, *p* = 0.045) and advanced PD patients (*p* = 0.028), whereas there was no significant difference between normal subjects and advanced PD patients (*p* = 0.69). The difference of temporal integration between early and advanced patients was not accounted for by LED since ANCOVA taking the LED as a covariate still proved to be significant (*p* = 0.021).

**Figure 2F** shows the time course of mean STE for the three subject groups when the ISI was 900 ms. As the tapping count increased, the tapping tended to be earlier, possibly reflecting the motor hastening. Early PD patients tended to tap earlier than normal subjects and advanced PD patients. The STE of advanced PD patients was less stable compared to other groups, possibly reflecting the motor symptom of fingers.

To see how the limit of temporal integration changed with disease progression, **Figure 2G** depicts the correlation between the UPDRS-III score and the limit of temporal integration. Multiple linear regression analysis demonstrated a trend toward negative correlation between the UPDRS-III score and the limit of temporal integration (*r* = -0.45, *p* = 0.081), in which the limit of temporal integration decreased with increasing UPDRS-III scores. On the other hand, none of the patient factors such as age, disease duration, and LED significantly correlated with the limit of temporal integration (**Table 3**).

**Table 3:** Results of multiple regression analysis of PD patients, where the outcome variable was the limit of temporal integration (ms).

Explanatory variable	*p-*value	β
Age (years)	0.99	0.91
Disease duration (years)	0.09	-196.4
Levodopa equivalent dose (mg/day)	0.34	2.38


To summarize, early PD patients tended to tap earlier than normal subjects (**Figure 2A**), and were less likely to lag behind the tones up to a certain ISI (**Figures 1B**, **2D**). This indicated a prolonged limit of temporal integration in early PD (**Figure 2E**). In contrast, advanced PD patients (UPDRS-III > 15) showed a shorter limit of temporal integration, even shorter than that of normal subjects, with only a small amount of negative asynchrony. Consequently, the limit of temporal integration tended to mildly decrease with advancing disease stage.

**Figures 2H,I** looks at the effect of L-dopa on the performance of this task. We plotted the UPDRS-III score of each subject both before (OFF) and after (ON) L-Dopa intake. L-Dopa intake significantly reduced the UPDRS-III score, as shown by the paired *t*-test (*p* < 0.001). **Figure 2I** plots the averaged STE against each ISI in the L-Dopa ON and OFF states. In contrast to the improved UPDRS score, there was no statistically significant difference between the L-Dopa ON and OFF states at any ISI, suggesting that L-Dopa has no effect on the strategic shift of the synchronized tapping task.

**Figure 2J** shows the time courses of mean STE when L-Dopa was ON and OFF. The time courses of STE when L-Dopa was ON or OFF was almost identical, initially delayed but gradually earlier, reaching plateau after around 40 taps.

## Discussion

Our study demonstrated that early PD patients (UPDRS-III ≤ 15) show a tendency toward negative asynchrony as well as a prolonged limit of temporal integration compared to normal subjects. This result was unexpected for us, since we had predicted that mild PD patients would perform worse than normal subjects in that they would show more taps lagging behind the tones at earlier ISIs compared to normal subjects, reflecting slower internal clocks or increased variability in tapping performance, hampering proper temporal synchrony and leading to delayed tapping. In advanced PD patients (UPDRS-III > 15), however, the number of taps lagging behind the tones increased as it did in normal subjects, eventually resulting in a shorter limit of temporal integration, as we had predicted. L-Dopa had no effect on the tapping performance (**Figures 2I,J**).

### Tendency Toward Early Tapping in Early PD Patients

The tendency toward early tapping in early PD patients in the synchronized tapping task is consistent with previous studies showing negative asynchrony. As described above, when ISIs are shorter than 1 s, normal subjects tend to tap 30–50 ms earlier than the tones despite the subjective impression of exact synchronization (Aschersleben and Prinz, 1995). This tendency was more pronounced in early PD patients (**Figures 2A,F**), and became more deviated from that of normal subjects as ISI increased (**Figure 2A**).

How does negative asynchrony emerge? According to the process model of synchronization as proposed by Vorberg and Hambuch (1984), when subjects try to keep pace with tones presented at regular intervals, they try to synchronize the taps with the tones mainly by two mechanisms. One mechanism is to adjust their tapping through a trial-by-trial error correction process, using the perceived discrepancy between the time of tapping and that of the tone in each trial. Another mechanism is to adjust their overall tapping rate to approximate the pace of the presented tones through a tempo generation process. Negative asynchrony is more prominent in PD patients than in normal subjects, as reported previously (Diedrichsen et al., 2003). Although the exact mechanism of the greater negative asynchrony remains unclear (Aschersleben and Prinz, 1995; O’Boyle et al., 1996; Diedrichsen et al., 2003), some studies have postulated that negative asynchrony in normal subjects results from the difference in the processing time needed to perceive the tap (afferent information of finger movement) and the tone (afferent auditory information) (Aschersleben and Prinz, 1995). More specifically, the finger movement is perceived as occurring later than the auditory information, and the subject copes with this by unconsciously tapping earlier than the tone, so that both the finger movements and the tones can be perceived as occurring simultaneously. Aschersleben and Prinz (1995) has suggested that this tendency is more pronounced in PD patients than in normal subjects, as they rely more heavily on the first mechanism of temporal adjustment.

An alternative and more parsimonious explanation is that the internal clocks of PD patients are ticking faster than normal. This would indicate an increased pace of the internal clock in mild PD patients, who accordingly tap faster than the tones in a repeated series. This would also result in PD patients tapping earlier than the next oncoming tone, and hence in negative asynchrony. With increasing ISI, this advancement in tapping would be expected to accumulate as shown in **Figure 2F**, but to reach a plateau at some range due to auditory feedback from the tones. Consistent with this view, **Figure 2A** also shows that PD patients tend to tap earlier than the tones, and that the discrepancy with normal subjects also tended to grow with increasing ISI. This tendency toward earlier tapping would actually work to prevent delayed tapping until the patients were entirely incapable of keeping pace with the tones, thus enabling a longer limit of temporal integration. The implication of this phenomenon is that the movements of PD patients can sometimes be faster than those of normal subjects, especially engaged in a repetitive motor action (Honma et al., 2016).

Whatever the cause of negative asynchrony, the tendency toward negative asynchrony would help prolong the limit of temporal integration at the early stages of PD, reducing the proportion of delayed responses. In advanced PD patients (black triangles), in contrast, the tendency toward negative asynchrony becomes much less apparent compared with normal subjects, and the curve plotting STE against ISI (**Figure 2A**) nearly overlapped with that of normal subjects. Thus, with the progression of PD, the tendency toward advanced tapping indicating a faster internal clock may have been lost as the internal clock eventually came to “tick” slower. Alternatively, temporal integration may be disrupted since the pace of the clock may become too variable to keep pace with the tones, i.e., to predict the time of the oncoming tone to prepare the next tapping with it.

### Faster Speed of Internal Clock and Its Association With Motor Symptoms in PD

The interpretation of a faster clock speed is at variance with the general view that the internal clock ticks at a slower pace in conditions of dopamine deficiency. In animal studies, for example, the dopamine D2 blocker haloperidol is known to prolong the production of time intervals toward a longer range (Buhusi and Meck, 2005). A slower clock speed makes sense because it is consistent with the general slowness in motor symptoms and cognitive slowing, also called bradyphrenia, in PD patients; this seems at first sight to rule out the possibility that the internal clock could be ticking faster than normal.

It is well known, however, that although PD patients are generally slow in their movement, they may also show motor hastening in some circumstances. For example, while they have difficulty initiating locomotion, once it is started their steps tend to be smaller and marked by faster cycle time. Such clinical symptoms could be interpreted as reflecting a bias for the internal clock to operate faster when engaged in a repetitive action. Likewise, the phenomenon of faster tapping with repetition may be linked to motor hastening in clinical neurology although the exact underlying mechanism might differ from that of hastening in tapping.

In fact, there are also suggestions that the internal clocks of PD patients may tick faster than those of normal subjects under some conditions. A similar hastening was observed in a study by Honma et al. (2016) in which PD patients were instructed to produce time intervals corresponding to the number of seconds shown on a screen by counting the seconds in the head. PD patients produced shorter time intervals than normal subjects did; in other words, PD patients underestimated the time interval. This underestimation is consistent with faster ticking of the internal clock. Honma et al. (2016) also showed that, when subjects tapped at an interval of their *subjective* 1 s in the simple tapping task, PD patients, but not normal subjects, gradually tapped faster and faster, again corresponding to a faster ticking of the internal clock in PD patients.

Taken together with the present results, this finding may indicate that the seemingly faster ticking of the internal clock occurs when it is engaged in a repetitive manner requiring temporal processing. Instead of reflecting a faster pace of the internal clock *per se*, PD patients perceive their movements to be slower than they actually are, and may compensate for this by moving faster (tapping faster) or by initiating movements earlier (negative asynchrony).

### Increase of Delayed Tapping in Advanced PD Patients

In contrast, why did the limit of temporal integration shorten in advanced PD? One possible interpretation would be that, as PD progresses, internally guided movements become impaired whereas externally guided movements remain largely intact (Praamstra et al., 1998). As a result, the preparation of movements in the absence of an external cue but in anticipation of a forthcoming event (in this case, the tone) becomes progressively impaired, whereas delayed responses to the cue, an externally guided movement, are relatively preserved and come to prevail over the former. On the other hand, whereas advanced PD patients may also lose the tendency toward negative asynchrony as shown in **Figure 2A**, with progression, the temporal prediction to synchronize with the oncoming tone would eventually break down or become too variable, such that tapping by advanced PD patients would tend to lag behind the tones, resorting to the “reactive mode” of tapping in response to the tones rather than attempting to tap in synchrony with or possibly ahead of the tones. This mechanism may have resulted in the increasing proportion of delayed tapping for ISIs longer than 2 s, and thus the shorter limit of temporal integration.

It may be argued that the increase in delayed tapping in advanced PD patients results from their prolonged reaction time, i.e., that it is a mere reflection of their motor symptoms. To test this, we studied the effect of L-Dopa on STE. If the increase in delayed tapping is a reflection of motor symptoms, it should be improved by taking L-Dopa. In fact, L-Dopa had no effect on the performance of synchronization (**Figure 2I**), although it improved the UPDRS-III scores of the patients (**Figure 2H**). Therefore, the increase in delayed tapping is unlikely to have resulted from the increased reaction time or the motor symptoms, as these were improved by L-dopa.

### Difference in Temporal Processing Between PD and Cerebellar Patients

As mentioned in the Introduction, the basal ganglia and the cerebellum have both come to be implicated in different aspects of temporal processing. Temporal processing on the subsecond order, which is implicated in the “automatic” or “bottom-up” system, is considered to be processed by the cerebellum, whereas suprasecond timing, which is important in “cognitively controlled” or “top-down” system processing, is processed by the prefrontal and parietal cortical regions as well as the basal ganglia (Ivry, 1996; Lewis and Miall, 2003; Meck, 2005; Hayashi et al., 2014).

To our knowledge, this is the first time that temporal integration has been explored in PD. In the supra- and infra-second contrast, we expected that temporal processing on the order of seconds, as studied by the limit of temporal integration in the range of 2–3 s, would be disrupted (shortened) in PD, which turned out to be true. It is important that we could show that the shortening of temporal integration was due to the abnormality in the rhythm generation, but disruption of the internal clock may also take place in the advanced stages of the disease, introducing a bias toward reactive tapping.

The performance of PD patients may be contrasted with that of cerebellar patients. Temporal integration has also been shown to be disrupted in cerebellar ataxia; Matsuda et al. (2015) showed that, in patients with spinocerebellar degeneration including SCA6 and SCA31, the temporal prediction required for the performance of this task is disrupted. Histograms of the kind shown here were made for normal subjects. Tapping performance varied around the time of tones (0 ms), but no negative asynchrony was evident (Matsuda et al., 2015). In cerebellar patients, on the other hand, STE was quite variable for all ISIs, showing that a great deal of noise affected their prediction of the times of oncoming tones. The resulting shortening was considered to be due primarily to the patients’ impaired ability to predict the times of forthcoming events. This trend was shown to occur regardless of the severity of spinocerebellar degeneration. The increased variability is believed to be due to their variable clocks, rather than the rhythm of internal clock, suggesting that a central role is played by the cerebellum in producing consistently timed responses at predicted times. However, the exact difference between these two categories of neurological disorders awaits further study.

As discussed in the section “Introduction,” in a PET study by Sadato et al. (1996), differential cortical and subcortical activation was observed with the synchronized tapping task at slower and faster tapping rates. Primary sensorimotor cortex contralateral to the tapping finger as well as the ipsilateral cerebellum showed no significant activation at slower tapping rates, but showed a rise with faster tapping rates. In contrast, the Supplementary Motor Area (SMA) as well as the cingulate cortex, prefrontal cortex and putamen, showed activation at slower tapping rates, but the activation declined for faster tapping rates. These progressive changes in activation pattern may reflect the change of CBF according to whether the task is performed with reactive and predictive modes. PD patients up to a certain stage of progression may be able to recruit the SMA as well as the basal ganglia necessary for the predicted tapping. However, this ability may be disrupted after a certain stage of disease has been reached due to basal ganglia dysfunction and the patients may have to resort more to the “reactive” mode of tapping, endorsing a bias for faster tapping rates, compensating the tapping performance by recruiting the primary sensorimotor cortex and cerebellum.

The limitations of the present study are that it is a behavioral study and that it does not reveal the precise underlying mechanisms of the observed phenomena at the neural level. In order to investigate the interactions between the cerebellar and basal ganglia systems in the synchronized tapping task and how they are affected in neurological disorders, future studies will be required to examine a whole range of patients with parkinsonism and cerebellar ataxia, to determine how the basal ganglia and cerebellum are affected to produce various extents and in various combinations of abnormalities, and to compare the performances of different patient types on the same ground.

Another limitation was that the number of subjects was small. Despite the small sample size, however, we could demonstrate a significant difference in the temporal integration between normal, early and advanced PD patients. Importantly, we could demonstrate a dissociation between early and advanced PD in tapping paradigm. Nevertheless, increasing the sample size in the future would enable us to further assess and characterize the temporal processing abnormalities in relation to the clinical symptoms of the patients (e.g., PD patients of postural instability and gait failure versus tremor dominant type).

Our research adds novel insights into the abnormality of temporal processing in PD patients, which has been relatively unexplored in past studies. Further characterization of the ability of temporal integration in various neurological disorders such as SCD could contribute to the understanding of the temporal processing abnormality in such disorders.

## Author Contributions

YT and S-IT planned the study and experiments. S-IT and SM performed the experiments. S-IT analyzed the data. S-IT and YT wrote the manuscript. TF, TS, SI-T, AY, MH, ST, and YU contributed to revise the manuscript.

## Conflict of Interest Statement

YU was supported by a Research Project Grant-in-aid for Scientific Research from the Ministry of Education, Culture, Sports, Science and Technology of Japan (25293206, 15H05881); by The Research Committee on Degenerative Ataxia from the Ministry of Health, Labour and Welfare of Japan; and by a grant from the Committee of the Study of Human Exposure to EMF from the Ministry of Public Management, Home Affairs, Post and Telecommunications; has received speaker’s honoraria from the Taiwan Movement Disorders Society, Astellas Pharma Inc., Eisai Co., Ltd., FP Pharmaceutical Corporation, Otsuka Pharmaceutical Co., Ltd., Elsevier Japan K.K., Kissei Pharmaceutical CO., Ltd., Kyorin Pharmaceutical Co., Ltd., Kyowa Hakko Kirin Co., Ltd., GlaxoSmithKline K.K., Sanofi-Aventis K.K., Daiichi Sankyo Co., Ltd., Dainippon Sumitomo Pharma Co., Ltd., Takeda Pharmaceutical Co., Ltd., Mitsubishi Tanabe Pharma Corporation, Teijin Pharma Limited, Nippon Chemiphar Co., Ltd., Nihon Pharmaceutical Co., Ltd., Nippon Boehringer Ingelheim Co., Ltd., Novartis Pharma K.K., Bayer Yakuhin, Ltd., and Mochida Pharmaceutical Co., Ltd.; and has received royalties from Chugai-Igakusha, Igaku-Shoin Ltd., Medical View Co. Ltd., and Blackwell Publishing K. K. YT was supported by a Research Project Grant-in-aid for Scientific Research from the Ministry of Education, Culture, Sports, Science and Technology of Japan (16H01497 and 16K09709), and by GlaxoSmithKline and Boehringer Ingelheim, and has received speaker’s honoraria from Boehringer Ingelheim. MH serves as a medical advisor for Pfizer Japan Inc. The remaining authors declare that the research was conducted in the absence of any commercial or financial relationships that could be construed as a potential conflict of interest.

## References

[B1] Aschersleben G. Prinz W. (1995). Synchronizing actions with events: the role of sensory information. *Percept. Psychophys.* 57 305–317. 10.3758/BF03213056 7770322

[B2] Buhusi C. V. Meck W. H. (2005). What makes us tick? Functional and neural mechanisms of interval timing. *Nat. Rev. Neurosci.* 6 755–765. 10.1038/nrn1764 16163383

[B3] Claassen D. O. Jones C. R. Yu M. Dirnberger G. Malone T. Parkinson M. (2013). Deciphering the impact of cerebellar and basal ganglia dysfunction in accuracy and variability of motor timing. *Neuropsychologia* 51 267–274. 10.1016/j.neuropsychologia.2012.09.018 23084982

[B4] Church R. M. Meck W. H. Gibbon J. (1994). Application of scalar timing theory to individual trials. *J. Exp. Psychol. Anim. Behav. Process.* 20 135–155. 10.1037/0097-7403.20.2.135 8189184

[B5] Diedrichsen J. Ivry R. Pressing J. (2003). “Cerebellar and basal ganglia contributions to interval timing,” in *Functional and Neural Mechanisms of Interval Timing*, ed. Meck W. H. (New York, NY: CRC Press), 457–483.

[B6] Drake C. Jones M. R. Baruch C. (2000). The development of rhythmic attending in auditory sequences: attunement, referent period, focal attending. *Cognition* 77 251–288. 10.1016/S0010-0277(00)00106-2 11018511

[B7] Fraisse P. (1984). Perception and estimation of time. *Annu. Rev. Psychol.* 35 1–36. 10.1146/annurev.ps.35.020184.000245 6367623

[B8] Gibbon J. Church R. M. Meck W. H. (1984). Scalar timing in memory. *Ann. N. Y. Acad. Sci.* 423 52–77. 10.1111/j.1749-6632.1984.tb23417.x 6588812

[B9] Harrington D. L. Haaland K. Y. (1999). Neural underpinnings of temporal processing: a review of focal lesion, pharmacological, and functional imaging research. *Rev. Neurosci.* 10 91–116. 10.1515/REVNEURO.1999.10.2.91 10658954

[B10] Hayashi M. J. Kantele M. Walsh V. Carlson S. Kanai R. (2014). Dissociable neuroanatomical correlates of subsecond and suprasecond time perception. *J. Cogn. Neurosci.* 23 1–9. 10.1162/jocn_a_00580 24456398

[B11] Hazeltine E. Helmuth L. L. Ivry R. B. (1997). Neural mechanisms of timing. *Trends Cogn. Sci.* 1 163–169. 10.1016/S1364-6613(97)01058-9 21223897

[B12] Honma M. Kuroda T. Futamura A. Shiromaru A. Kawamura M. (2016). Dysfunctional counting of mental time in Parkinson’s disease. *Sci. Rep.* 6:25421. 10.1038/srep25421 27146904 PMC4857080

[B13] Ivry R. B. (1996). The representation of temporal information in perception and motor control. *Curr. Opin. Neurobiol.* 6 851–857. 10.1016/S0959-4388(96)80037-7 9000026

[B14] Jones C. R. Jahanshahi M. (2009). The substantia nigra, the basal ganglia, dopamine and temporal processing. *J. Neural Transm. Suppl.* 73 161–171. 10.1007/978-3-211-92660-4_13 20411776

[B15] Jones M. R. (1976). Time, our lost dimension: toward a new theory of perception, attention, and memory. *Psychol. Rev.* 83 323–355. 10.1037/0033-295X.83.5.323 794904

[B16] Koch G. Costa A. Brusa L. Peppe A. Gatto I. Torriero S. (2008). Impaired reproduction of second but not millisecond time intervals in Parkinson’s disease. *Neuropsychologia* 46 1305–1313. 10.1016/j.neuropsychologia.2007.12.005 18215403

[B17] Lange K. W. Tucha O. Steup A. Gsell W. Naumann M. (1995). Subjective time estimation in Parkinson’s disease. *J. Neural Trans. Suppl.* 46 433–438. 8821079

[B18] Lewis P. A. Miall R. C. (2003). Brain activation patterns during measurement of sub- and supra-second intervals. *Neuropsychologia* 41 1583–1592. 10.1016/S0028-3932(03)00118-0 12887983

[B19] Mates J. Müller U. Radil T. Pöppel E. (1994). Temporal integration in sensorimotor synchronization. *J. Cogn. Neurosci.* 6 332–340. 10.1162/jocn.1994.6.4.332 23961729

[B20] Matsuda S. Matsumoto H. Furubayashi T. Hanajima R. Tsuji S. Ugawa Y. (2015). The 3-second rule in hereditary pure cerebellar ataxia: a synchronized tapping study. *PLoS One* 10:e0118592. 10.1371/journal.pone.0118592 25706752 PMC4337906

[B21] Meck W. H. (2005). Neuropsychology of timing and time perception. *Brain Cogn.* 58 1–8. 10.1016/j.bandc.2004.09.004 15878722

[B22] Nenadic I. Gaser C. Volz H. P. Rammsayer T. Häger F. Sauer H. (2003). Processing of temporal information and the basal ganglia: new evidence from fMRI. *Exp. Brain Res.* 148 238–246. 10.1007/s00221-002-1188-4 12520413

[B23] O’Boyle D. J. Freeman J. S. Cody F. W. (1996). The accuracy and precision of timing of self-paced, repetitive movements in subjects with Parkinson’s disease. *Brain* 119 51–70. 10.1093/brain/119.1.51 8624694

[B24] Pastor M. A. Artieda J. Jahanshahi M. Obeso J. A. (1992). Time estimation and reproduction is abnormal in Parkinson’s disease. *Brain* 115 211–225. 10.1093/brain/115.1.211 1559155

[B25] Perbal S. Deweer B. Pillon B. Vidailhet M. Dubois B. Pouthas V. (2005). Effects of internal clock and memory disorders on duration reproductions and duration productions in patients with Parkinson’s disease. *Brain Cogn.* 58 35–48. 10.1016/j.bandc.2005.02.003 15878725

[B26] Pöppel E. (1978). “Time perception,” in *Handbook of Sensory Physiology, Perception*, Vol. 8 eds Held R. Leibowitz H. W. Teuber H. L. (Berlin: Springer), 713–729.

[B27] Pöppel E. (1997). Hierarchical model of temporal perception. *Trends Cogn. Sci.* 1 56–61. 10.1016/S1364-6613(97)01008-5 21223864

[B28] Praamstra P. Stegeman D. F. Cools A. R. Horstink M. W. (1998). Reliance on external cues for movement initiation in Parkinson’s disease. Evidence from movement-related potentials. *Brain* 121 167–177. 10.1093/brain/121.1.167 9549497

[B29] Rao S. M. Harrington D. L. Haaland K. Y. Bobholz J. A. Cox R. W. Binder J. R. (1997). Distributed neural systems underlying the timing of movements. *J. Neurosci.* 17 5528–5535. 10.1523/JNEUROSCI.17-14-05528.1997 9204934 PMC6793838

[B30] Repp B. H. (2005). Sensorimotor synchronization: a review of the tapping literature. *Psychon. Bull. Rev.* 12 969–992. 10.3758/BF03206433 16615317

[B31] Sadato N. Ibañez V. Deiber M. P. Campbell G. Leonardo M. Hallett M. J. (1996). Frequency-dependent changes of regional cerebral blood flow during finger movements. *J. Cereb. Blood Flow Metab.* 16 23–33. 10.1097/00004647-199601000-00003 8530552

[B32] Schubotz R. I. Friederici A. D. von Cramon D. Y. (2000). Time perception and motor timing: a common cortical and subcortical basis revealed by fMRI. *Neuroimage* 11 1–12. 10.1006/nimg.1999.0514 10686112

[B33] Takano K. Miyake Y. (2007). Two types of phase correction mechanism involved in synchronized tapping. *Neurosci. Lett.* 417 196–200. 10.1016/j.neulet.2007.02.044 17395371

[B34] Tolleson C. M. Dobolyi D. G. Roman O. C. Kanoff K. Barton S. Wylie S. A. (2015). Dysrhythmia of timed movements in Parkinson’s disease and freezing of gait. *Brain Res.* 1624 222–231. 10.1016/j.brainres.2015.07.041 26241766 PMC4630160

[B35] von Steinbüchel N. Wittmann M. Pöppel E. (1996). “Timing in perceptual and motor tasks after disturbances of the brain,” in *Time, Internal Clocks and Movement*, eds Pastor M. A. Artieda J. (Amsterdam: Elsevier), 281–304. 10.1016/S0166-4115(96)80064-1

[B36] Vorberg D. Hambuch R. (1984). “Timing of two-handed rhythmic performance,” in *Annals of the New York Academy of Sciences, Timing and Time Perception* Vol. 423 eds Gibbon L. Allan L. (New York, NY: New York Academy of Sciences), 390–406. 10.1111/j.1749-6632.1984.tb23448.x 6588803

[B37] Wing A. Kristofferson A. (1973). Response delays and the timing of discrete motor responses. *Percept. Psychophys.* 14 5–12. 10.3758/BF03198607 11543514

[B38] Yahalom G. Simon E. S. Thorne R. Peretz C. Giladi N. (2004). Hand rhythmic tapping and timing in Parkinson’s disease. *Parkinsonism Relat. Disord.* 10 143–148. 10.1016/j.parkreldis.2003.10.001 15036168

